# Identification of novel linear epitopes in P72 protein of African swine fever virus recognized by monoclonal antibodies

**DOI:** 10.3389/fmicb.2022.1055820

**Published:** 2022-11-02

**Authors:** Dan Yin, Renhao Geng, Hongxia Shao, Jianqiang Ye, Kun Qian, Hongjun Chen, Aijian Qin

**Affiliations:** ^1^The International Joint Laboratory for Cooperation in Agriculture and Agricultural Product Safety, Ministry of Education, Yangzhou University, Yangzhou, China; ^2^Jiangsu Co-Innovation Center for Prevention and Control of Important Animal Infectious Diseases and Zoonoses, Yangzhou, China; ^3^Jiangsu Key Laboratory of Zoonosis, Yangzhou, China; ^4^Shanghai Veterinary Research Institute, Chinese Academy of Agricultural Sciences (CAAS), Shanghai, China

**Keywords:** African swine fever virus, monoclonal antibodies, p72, B602L, epitope

## Abstract

African swine fever (ASF) is one of the highly contagious and lethal diseases among domestic pigs and wild boars. The capsid protein P72 of African swine fever virus (ASFV) is very important for the diagnosis and vaccine development. However, the epitope of the protein is not clear. In this study, capsid protein P72 was expressed in Sf9 cells along with its chaperone B602L. A total of ten monoclonal antibodies (mAbs) specific to P72 protein were developed by fusions between SP2/0 cells and spleen cells of mice immunized with the recombinant-P72&B602L proteins expressed in Sf9 cells. Four linear B cell epitopes ^31^SNIKNVNKSY^40^, ^41^GKPDP^45^, ^56^HLVHFNAH^63^ and ^185^ERLYE^189^ were identified. Biological information analysis illustrated that epitopes ^31^SNIKNVNKSY^40^, ^41^GKPDP^45^ and ^185^ERLYE^189^ were highly conserved within different ASFV strains. These findings may lead to a better understanding of the antibody-antigen interaction and provide new insights into the vaccine research and serological diagnosis of ASF.

## Introduction

African swine fever (ASF) is caused by African swine fever virus (ASFV), a highly complex, large, and enveloped DNA virus belonging to the genus Asfivirus, family Asfarviridae (Alonso et al., 2018). The disease is highly contagious and lethal, usually causing up to 100% mortality in domestic pigs, and is classified as a notifiable disease by the World Organization for Animal Health (OIE) (Galindo and Alonso, 2017; Zhao et al., 2019).

African swine fever virus has a complex structure with multiple membranes and protein layers (Revilla et al., 2018). The outmost protein coat of the virion is an icosahedral capsid, which is mainly assembled from the protein P72 encoded by virus gene *B646L*. The major capsid protein (MCP) P72 is the most important structural component of the virion, accounting for about one-third of the total weight of the virus particle, making it one of the major antigens detected in infected pigs (Kollnberger et al., 2002; Yang et al., 2021). One of ASF control strategies is the early detection for the infected pigs, preventing viral spread and prompting eradication in advance (Dixon et al., 2020). Currently, control strategies rely on nucleic acid, antigen and antibody detections. The MCP P72 is considered to be an ideal antigen for routine serologic diagnosis due to its immunogenicity and antigen stability (Kollnberger et al., 2002). However, this application is sometimes haltered by the insolubility of recombinant P72 protein due to possible conformation mis-presentation (Yu et al., 1996). Therefore, different strategies ought to be applied for the aim of producing more reliable P72 antigens and antibodies. Early studies showed that another protein, B602L, is required for the formation of the viral icosahedral capsid and increasing trypsin-resistant P72 output (Liu et al., 2019). B602L has been described as a molecular chaperone for the correct folding of the MCP P72 (Epifano et al., 2006).

The P72 protein contains conserved immunogenic regions, suggesting that the P72 protein can potentially be used as a target to develop ASFV P72 mAbs for the detection of a broad spectrum of ASFV. Epitope identification of P72 is a key step in epitope-driven subunit vaccine design and immunodiagnostic tests. Although anti-P72 mAbs have been successfully produced, corresponding epitopes are limited. A single conformational neutralizing epitope and 4 linear epitopes have been identified on P72, but information on the other antigenic regions (epitopes) is absent (Borca et al., 1994; Heimerman et al., 2018).

In this study, we developed recombinant baculoviruses that co-express ASFV P72 and B602L, enabling correct expression of the P72 protein. A panel of mAbs against P72 recombinant protein were generated, together with their corresponding core linear B cell epitopes. Also, P72 sequences from different ASFV strains were aligned to analyze their relative conservation. These findings will be valuable for the development of epitope-based diagnostic kits and prophylactic techniques for ASFV infection.

## Materials and methods

### Virus and cells

African green monkey cells MA-104, human embryonic kidney 293T (HEK293T) cells and myeloma cell line SP2/0 stored in our laboratory were maintained in Dulbecco’s modified Eagle medium (DMEM) (Thermo Fisher Scientific, Massachusetts, USA) supplemented with 10% fetal bovine serum (FBS) (Thermo Fisher Scientific, Massachusetts, USA) in a humidified incubator with 5% CO_2_ at 37°C. The Spodoptera frugiperda insect cells (Sf9), stored in our laboratory, were cultured in Sf-900 II SFM medium (Gibco, USA) at 27°C. Cryopreservation of all cell lines were using CELLSAVING (New Cell & Molecular Biotech, China). The ASFV genome (GenBank accession number MH766894), ASFV-positive sera (Convalescent sera from surviving pigs naturally infected with ASFV) were kindly provided by professor Rongliang Hu (Institute of Changchun Veterinary Medicine, Jilin, China). The recombinant virus ASFVGZΔMGF100-1R (The open reading frame of MGF100-1R was replaced by an EGFP expression cassette) was prepared in the previous study (Liu et al., 2021).

### Construction of recombinant P72 and B602L baculoviruses

The fragments of *B646L* (encoding P72) and *B602L* genes were amplified from the ASFV genome (GenBank accession number MH766894) with specific primers (Supplementary Table 1) and cloned into the baculovirus transfer vector pFastBac HTA to construct the recombinant P72 and B602L baculoviruses vectors, namely pFast-P72 and pFast-B602L, respectively. These two vectors encode the target proteins with hexa-histidine tag (His-tag). Subsequently, the constructs were transformed into *E. coli* DH5α competent cells (Vazyme Biotech Co., Ltd., Nanjing, China), and confirmed by Sanger sequencing (Sangon Biotech, Shanghai, China).

According to Bac-to-Bac Expression System, the recombinant plasmids were transformed into DH10Bac *E. coli* cells to construct recombinant baculoviruses. The recombinant virus DNA, bacmid-P72 and bacmid-B602L, were extracted by alkaline process (Haberl et al., 2013). Then the recombinant virus DNA bacmid-P72 and/or bacmid-B602L were transfected into Sf9 cells. Five days post-infection, the supernatant and cells were collected for virus passage. After three passages of infection, the baculoviruses were harvested, and named as recombinant-P72 baculovirus, recombinant-B602L baculovirus and recombinant-P72&B602L baculovirus, respectively.

Sf9 cells were infected with recombinant-P72 baculovirus and/or recombinant-B602L baculovirus at 1-2 multiplicity of infections (MOI), or infected with recombinant-P72&B602L baculovirus at 2 MOI. 5 days post-infection, cells were fixed with 60% acetone and subjected to immunofluorescence assays (IFA) with ASFV-positive sera and fluorescein isothiocyanate-conjugated (FITC-conjugated) goat anti-swine IgG (Jackson, USA). Then, the expressed protein was also confirmed by western blotting with 6x-His Tag mAb (Thermo Fisher Scientific, USA) and horseradish peroxidase-conjugated (HRP-conjugated) goat anti-mouse IgG (Jackson, USA) using the Super ECL Detection Reagent (Cat No. 36208; Yeasen, Shanghai, China). Universal antibody diluent was purchased from New Cell & Molecular Biotech (NCM, China).

### Production and characterization of monoclonal antibodies against P72 protein

Monoclonal antibodies against P72 protein were produced according to the previous method (Tesfagaber et al., 2021). Briefly, the recombinant-P72&B602L expression proteins were emulsified with complete Freund’s adjuvant (Sigma-Aldrich, St. Louis, MO, USA) at a 1:1 ratio. 6-week-age BALB/c mice were immunized with 100 μg of immunogen. Three booster immunizations were performed every two weeks with the same dosage of immunogen emulsified with incomplete Freund’s adjuvant (Sigma-Aldrich, St. Louis, MO, USA). Three days post the fourth immunization, spleen cells were harvested and fused with SP2/0 cells. The hybridoma cells were selected in a hypoxanthine-aminopterin-thymidine (Sigma-Aldrich, St. Louis, MO, USA) and hypoxanthine-thymidine (Sigma-Aldrich, St. Louis, MO, USA) medium. The supernatants of hybridomas were screened for P72-specific antibodies by IFA on MA104 cells infected with ASFVGZΔMGF100-1R, which was performed under biosecurity level 3 (BSL-3) conditions in the Spirit Jinyu Biological Pharmaceutical Co. LTD. (Liu et al., 2021). Rhodamine red-conjugated goat anti-mouse IgG (Jackson, USA) was used as the secondary antibody in IFA analysis. Positive clones were amplified and subcloned to produce monoclonal hybridoma cell lines and mAbs. Antibody subtypes were determined through the SBA Clonotyping System-HRP kit (Southern Biotech, Birmingham, USA) according to the manufacturer’s instructions. The specificity of the mAbs against the recombinant protein and virions was evaluated using western blotting and IFA.

### Mapping of the linear B cell epitopes of P72 protein

Immunofluorescence assays and western blotting methods were used to identify the smallest epitope recognized by mAbs. The truncated *B646L* gene fragments were ligated into the vectors pCAGGS and transfected into 293T cells. A progressive procedure was adopted to identify the epitope recognized by each mAb, based on gradually shortened P72 truncated fragments, which was confirmed by IFA. A total of 50 truncated fragments (P1-P8) were designed and screened, and the length and position of these truncated fragments of P72 were shown in Figure 5. According to IFA results, partial truncated fragments of P72 were ligated into pET-32a and transformed into *E. coli* BL21 (DE3) cells (Vazyme Biotech Co., Ltd., Nanjing, China) for expression. The minimal epitopes recognized by the mAbs were further verified by western blotting. All primers were synthesized by Sangon Biotech (Shanghai, China) and listed in Supplementary Table 1.

**FIGURE 1:**
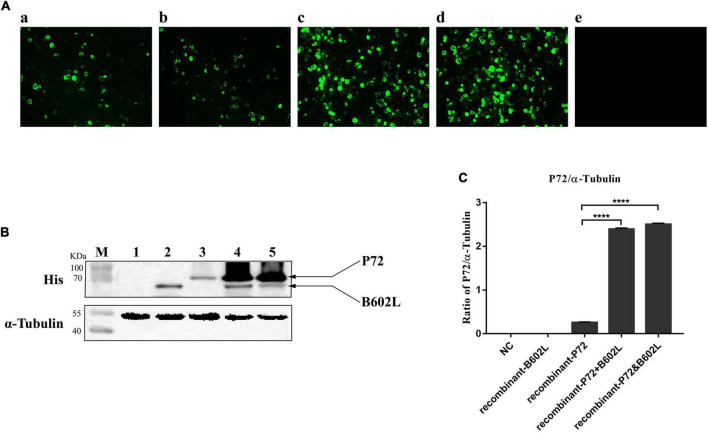
Immunofluorescence assays (IFA) and western blot analysis of Sf9 cells infected with recombinant baculovirus. **(A)** Indirect immunofluorescence staining of Sf9 cells infected with recombinant-B602L baculovirus (2 MOI) **(a)**, recombinant-P72 baculovirus (2 MOI) **(b)**, recombinant-P72&B602L baculovirus (2 MOI) **(c)**, or co-infected with recombinant-P72 (1 MOI) and recombinant-B602L baculovirus (1 MOI) **(d)**. Uninfected Sf9 cells were used as negative control **(NC) (e)**. The cells were fixed 5 days after infection and stained with the ASFV-positive sera and FITC-conjugated second antibodies. **(B)** Western blot analysis of crude lysates from Sf9 cells infected with recombinant-B602L baculovirus (2 MOI) (lane 2), recombinant-P72 baculovirus (2 MOI) (lane 3), co-infected with recombinant-P72 (1 MOI) and recombinant-B602L baculovirus (1 MOI) (lane 4), or recombinant-P72&B602L baculovirus (2 MOI) (lane 5). Uninfected Sf9 cells were used as NC (lane 1). As an internal control, α-tubulin was detected with anti-α-tubulin. M, protein molecular weight marker. **(C)** Relative P72 to α-tubulin ratios are quantified by Image J software. The student’s *t*-test compared two data sets marked by stars in the panel. Error bars represent the SD. The asterisks in the figures indicate significant differences (****P < 0.0001).

**FIGURE 2:**
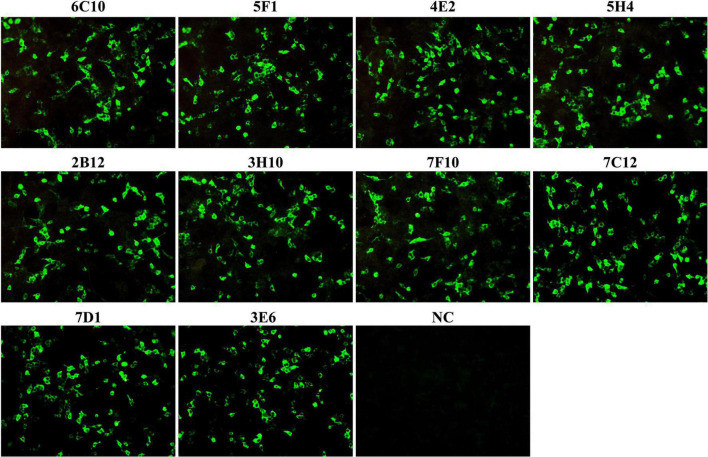
The reactivity of monoclonal antibodies (mAbs) was analyzed with immunofluorescence assays (IFA). HEK-293T cells were co-transfected with the plasmids pCAGGS-P72 and pCAGGS-B602L. Cells were fixed and stained with anti-P72 mAbs (6C10, 5F1, 4E2, 5H4, 2B12, 3H10, 7F10, 7C12, 7D1, and 3E6) as the primary antibody and FITC-conjugated goat anti-mouse IgG as the secondary antibody. Following staining, all anti-P72 mAbs exhibited green fluorescence. NC was HEK 293T cells stained with cell supernatants from SP2/0 cells.

**FIGURE 3:**
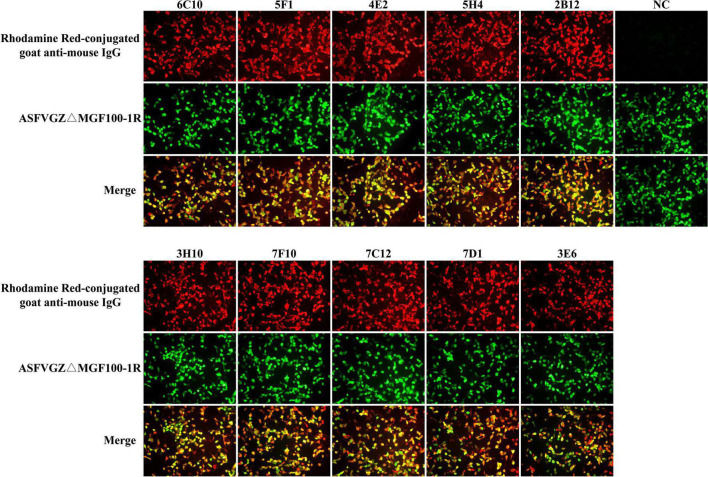
Reactivity of monoclonal antibodies (mAbs) to African swine fever virus (ASFV)-infected cells in immunofluorescence assays (IFA). MA104 cells were infected with the recombinant ASFVGZΔMGF100-1R deleted mutant showing EGFP expression **(green)**. Cells were fixed and incubated with anti-P72 mAbs (6C10, 5F1, 4E2, 5H4, 2B12, 3H10, 7F10, 7C12, 7D1, and 3E6) as indicated and stained with Rhodamine Red-conjugated goat anti-mouse IgG **(red)**. Cell supernatant of SP2/0 was used as primary antibody in the NC group.

**FIGURE 4:**
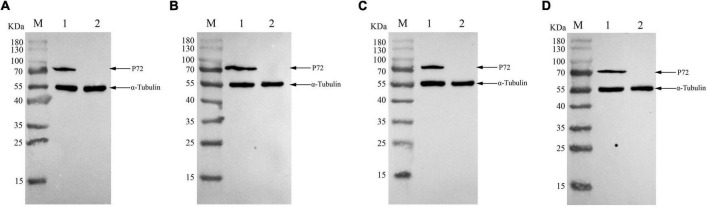
Reactivity of monoclonal antibodies (mAbs) to African swine fever virus (ASFV)-infected cells in western blotting. ASFVGZΔMGF100-1R infected MA104 cells were harvested at 48h post-infection, and western blotting analysis was performed using anti-P72 mAbs 6C10 **(A)**, 5F1 **(B)**, 4E2 **(C)** and 5H4 **(D)**. The size of the target protein was estimated to be 72 KDa. M, protein molecular weight marker; lane 1, ASFVGZΔMGF100-1R infected MA104 cells; lane 2, mock-infected MA104 cells. As an internal control, α-tubulin was detected with anti-α-tubulin.

**FIGURE 5:**
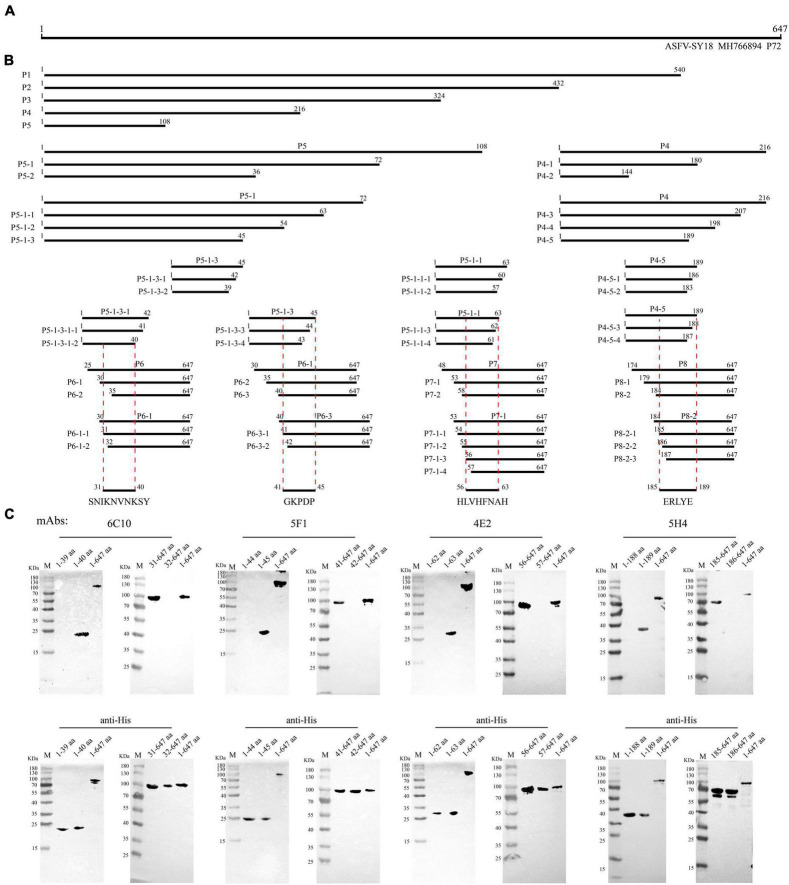
Schematic diagram of epitope mapping. **(A)** Full-length P72 protein of African swine fever virus (ASFV). **(B)** Design of truncated fragments (P1-P8) in P72 protein and the position of the four epitopes (^31^SNIKNVNKSY^40^, ^41^GKPDP^45^, ^56^HLVHFNAH^63^ and ^185^ERLYE^189^). **(C)** List of mAbs reacting with the corresponding P72 regions and precise mapping of mAbs epitopes with western blot. aa is an abbreviation for amino acid.

### Biological information analysis

The conservation analysis of the identified epitopes was implemented by Clustal W in MegAlign software, version 7.1.0 (DNAstar). The spatial distribution and the structure of the identified epitopes within P72 (PDB: 6L2T) were visualized by the PyMOL Molecular Graphics System (Version 2.4.0, Schrödinger, LLC.).

## Results

### B602L protein promotes the expression of P72 protein in Sf9 cells

To examine whether co-expression of P72 with B602L leads to an increase in the output of protein P72, Sf9 cells infected with recombinant baculoviruses expressing ASFV P72 and/or B602L were harvested 5 days post-infection. IFA results showed that Sf9 cells infected with only recombinant-P72 baculovirus or recombinant-B602L baculovirus generated poor signals, while those infected with recombinant-P72&B602L baculovirus or co-infected with both recombinant-P72 and recombinant-B602L baculoviruses displayed strongly with ASFV-positive sera (Figure 1A). At the same time, western blotting analysis was carried out with anti-His-tag mAb. The target protein reacted with the anti-His-tag mAb but not with the negative control. When P72 and B602L were expressed simultaneously in Sf9 cells, the expression level of P72 was significantly increased (*P* < 0.0001; Figures 1B,C). These results proved that co-expression of P72 and B602L contributed to the production and assembly of P72.

### Development of monoclonal antibodies against P72 protein

To produce anti-P72 mAbs, mice were immunized with the recombinant-P72&B602L proteins. 10 hybridoma cell lines producing antibodies to P72 were obtained, namely 6C10, 5F1, 4E2, 5H4, 2B12, 3H10, 7F10, 7C12, 7D1, and 3E6, respectively. These mAbs belonged to two subtypes: IgG1 and IgG2a (Table 1). All the mAbs showed positive IFA reactions in HEK-293T cells co-transfected with pCAGGS-P72 and pCAGGS-B602L (Figure 2) as well as ASFVGZΔMGF100-1R infected MA104 cells (Figure 3). Notably, 3 mAbs (7F10, 7C12 and 7D1) showed negative IFA reactions in HEK-293T cells transfected with pCAGGS-P72 or pCAGGS-B602L alone (Table 1). This finding suggested that mAbs against novel epitopes of P72 can be obtained with the help of B602L. Results from western blotting showed that 4 mAbs (6C10, 5F1, 4E2 and 5H4) reacted strongly with the denatured P72 protein, suggesting that these mAbs recognized mainly linear epitopes (Figure 4). The IFA and western blotting reactions of the 10 mAbs are also listed in Table 1.

**TABLE 1:** Reactivity of monoclonal antibodies (mAbs) to African swine fever virus (ASFV) P72 protein.

mAb	Isotype	ASFVGZΔMGF100-1R		HEK-293T cells co-transfected		HEK-293T cells	HEK-293T cells
		infected MA104 cells		with pCAGGS-P72 and pCAGGS-B602L		transfected with pCAGGS-P72	transfected with pCAGGS-B602L
				
		IFA	WB	IFA	WB	IFA	IFA
6C10	lgG1- Kappa	+	+	+	+	+	−
5F1	lgG1- Kappa	+	+	+	+	+	−
4E2	lgG1- Kappa	+	+	+	+	+	−
5H4	lgG1- Kappa	+	+	+	+	+	−
2B12	lgG1- Kappa	+	−	+	−	+	−
3H10	lgG1- Kappa	+	−	+	−	+	−
7F10	lgG1- Kappa	+	−	+	−	−	−
7C12	lgG1- Kappa	+	−	+	−	−	−
7D1	IgG2a- Kappa	+	−	+	−	−	−
3E6	IgG2a- Kappa	+	−	+	−	+	−

(IFA) immunofluorescence assays; (WB) western blot; (+) positive; (−) negative.

### Epitope mapping of anti-P72 monoclonal antibodies

To map the epitopes of 4 mAbs, 50 truncated P72 fragments expressed in HEK293T cells were used to investigate their reactions with antibodies. The length and position of these truncated fragments in P72 are shown in Figures 5A,B. The results of 4 mAbs’ reactivity were summarized in Supplementary Table 2. Next, the minimal linear epitopes were verified by western blotting. To determine the minimal residues of the linear B cell epitopes, the fragments P5-1-3-1 (1-42 aa), P5-1-3 (1-45 aa), P5-1-1 (1-63 aa), and P4-5 (1-189 aa) were further truncated from C-terminus by deleting amino acids one by one, and the fragments P6-1 (30-647 aa), P6-3 (40-647 aa), P7-1 (53-647 aa), and P8-2 (184-647 aa) were further truncated from N-terminus by deleting an amino acid one time. For P5-1-3-1, C-truncated fragments bound mAb 6C10 effectively until the ^40^Tyr residue was removed (Figure 5C). For P6-1, N-truncated fragments bound 6C10 strongly until the ^31^Ser residue was removed (Figure 5C). This indicated that peptide ^31^SNIKNVNKSY^40^ was the minimal residue. For P5-1-3 and P6-3, the binding capability of truncated fragments was completely lost when ^41^Gly or ^45^Pro was removed (Figure 5C), indicating that the minimal residue required by mAb 5F1 was ^41^GKPDP^45^. For P5-1-1 and P7-1, the binding capability of truncated fragments was completely lost when ^56^His or ^63^His was removed (Figure 5C), indicating that the minimal residue required by mAb 4E2 was ^56^HLVHFNAH^63^. For P4-5, the mAb 5H4 effectively recognized the C-truncated fragments until the deletion of ^189^Glu (Figure 5C). Moreover, the deletion of ^185^Glu led to the loss of the immunoreactivity of the N-truncated fragments P8-2 (Figure 5C). The results indicated that the minimal residue required by mAb 5H4 was ^185^ERLYE^189^. In summary, the 10-amino acid epitope (^31^SNIKNVNKSY^40^) for 6C10, the five-amino acid epitope (^41^GKPDP^45^) for 5F1, the nine-amino acid epitope (^56^HLVHFNAH^63^) for 4E2 and five-amino acid epitope (^185^ERLYE^189^) for 5H4 were minimally required for antibody-antigen interactions.

### Biological information analysis of the identified epitopes

**FIGURE 6:**
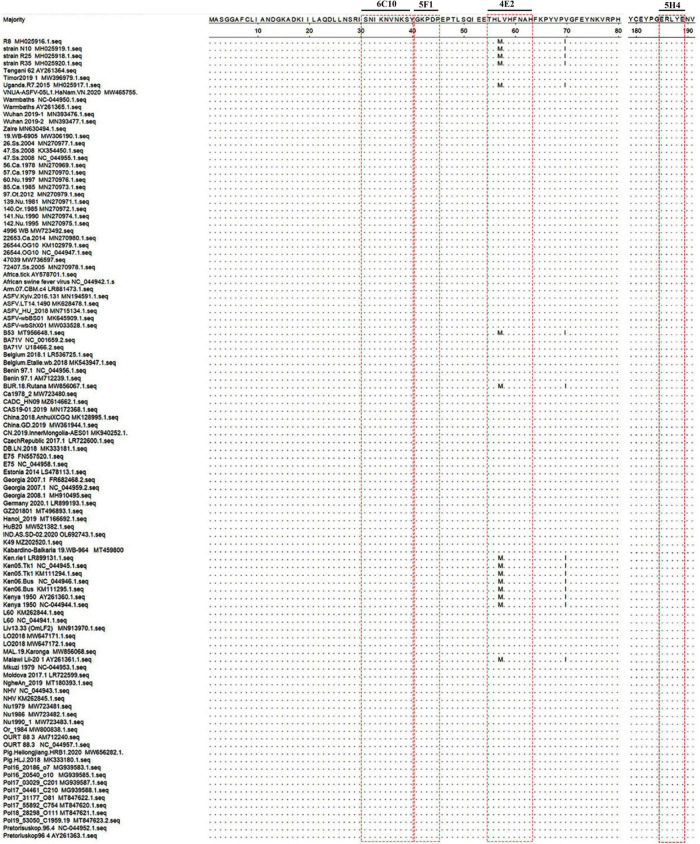
Conservation and location of the identified epitopes. Alignment of P72 protein sequences of different African swine fever virus (ASFV) strains was shown. Only the different residues were shown. The red box indicated the identified epitopes.

**FIGURE 7:**
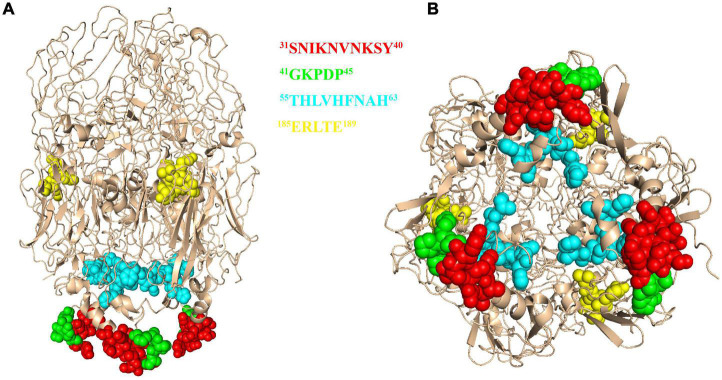
The 3D model of African swine fever virus (ASFV) P72 protein. The epitope ^31^SNIKNVNKSY^40^ was marked in red, epitope ^41^GKPDP^45^ was marked in green, epitope ^56^HLVHFNAH^63^ was marked in cyan and epitope ^185^ERLYE^189^ was marked in yellow. The structure is shown in the front view **(A)** and bottom view **(B)**.

To further analyze the conservation of the identified epitopes among different ASFV strains, sequences of 106 strains that represented the current status of epidemics worldwide were downloaded from GenBank, and their P72 sequences were aligned by MegAlign software. No amino acid substitutions and gaps were observed within ^31^SNIKNVNKSY^40^, ^41^GKPDP^45^ and ^185^ERLYE^189^, indicating that the three epitopes were highly conserved among ASFV prevailing strains (Figure 6). In the epitope ^56^HLVHFNAH^63^, an amino acid substitution (L→M) at position 57 was observed in multiple sequences. The result implied that the epitope ^56^HLVHFNAH^63^ might be less conserved than the other three epitopes. To understand the spatial distribution of the identified epitopes, the 3D model of P72 (PDB: 6L2T) was used for further analysis. As shown in Figure 7, the linear epitope ^185^ERLYE^189^ is located in the middle of the trimer and the remaining three linear epitopes ^31^SNIKNVNKSY^40^, ^41^GKPDP^45^ and ^56^HLVHFNAH^63^ are all located at the pseudo hexagonal base of the trimer.

## Discussion

In the past 100 years, ASF has emerged as a major threat to the global pig industry. Recently, ASF outbreaks in China, Southeast Asia and Belgium have created a sense of urgency to develop effective ways against ASF from entering negative countries (Zhou et al., 2018; Garigliany et al., 2019; Nga et al., 2020). No antiviral drug is available to efficiently prevent or treat ASF nowadays. Due to the complex nature of the virus and many undefined features of ASFV immunobiology, developing effective ASF vaccines remain a challenge (Teklue et al., 2020). Epitope information about this virus is crucial because it is increasingly being used for the rational design of vaccines and to distinguish mAbs (Palatnik-de-Sousa et al., 2018). The P72 protein, which is critical in the formation of major components of the viral capsid, is involved in viral entry and plays an important role in the viral attachment (Gomez-Puertas et al., 1996). However, there is relatively little information on the exact epitope of P72. Here, we provided new insight into epitope mapping of P72 protein.

P72 is also a good candidate for antibody detection and considered to be one of the most immunogenic proteins (Yu et al., 1996). According to previous studies, P72 was expressed as insoluble inclusion bodies in the *E. coli* expression system (Freije et al., 1993). Furthermore, Wan et al. have developed a colloidal-gold dual immunochromatographic strip with truncated P72 using the *E. coli* expression system (Wan et al., 2022). In addition, the truncated P72 is expressed independently in the baculovirus expression system for mAb production (Heimerman et al., 2018). It was demonstrated, however, that the p72 is unable to form trimers if expressed alone (Cobbold et al., 2001). In fact, the non-structural protein B602L has been described as a chaperone facilitating the correct folding of the p72 (Cobbold et al., 2001; Epifano et al., 2006). Previous studies have shown that only when P72 and B602L were co-expressed in HEK293F cells, correctly folded and assembled P72 can be obtained, and a highly specific colloidal gold immunochromatographic strip was developed based on this (Liu et al., 2019; Geng et al., 2022). The conformational misrepresentation of recombinant P72 protein may obscure some important epitopes and limit the production of mAbs. One advantage of the baculovirus system is the production of proteins with post-translational modifications. Therefore, we have created a new generation of recombinant baculoviruses that co-express ASFV P72 and B602L, thereby promoting the correct folding and assembly of the P72 protein. A significant increase in P72 expression was observed when P72 and B602L were co-expressed in Sf9 cells (Figure 1), consistent with the previous study in HEK293F cells (Liu et al., 2019). These findings may provide novel insights into ASFV vaccine development.

Next, 10 anti-P72 mAbs were developed based on recombinant baculoviruses co-expressing ASFV P72 and B602L. At the same time, 2 anti-B602L mAbs named 1F3 and 2E11 were also obtained in this experiment. And further studies on the characterization of anti-B602L mAbs are under process. Interestingly, 3 of the 10 anti-P72 mAbs in the IFA assay reacted well with HEK-293T cells co-transfected with pCAGGS-P72 and pCAGGS-B602L, but not with pCAGGS-P72 or pCAGGS-B602L transfected alone (Table 1). Therefore, in this study, three new anti-P72 mAbs (7F10, 7C12 and 7D1) recognized conformational epitopes of P72 protein that can be formed only in the presence of B602L. In addition, during the screening of mAbs against ASFV P72 protein, we found that mAbs with conformational epitopes had more dominance. These results suggested that B602L protein can assist the correct assembly and folding of P72 protein in Sf9 cells. Meanwhile, the chaperone B602L can help us screen mAbs against some important epitopes.

We found that four mAbs (6C10, 5F1, 4E2, and 5H4) reacted with denatured protein P72, indicating that these mAbs can be used for linear epitope identification. Epitopes are important factors in determining the antigenicity of viral structural proteins and influencing humoral immunity (Corral-Lugo et al., 2020). Identification of ASFV B-cell epitopes can help understand virus-host interactions and is essential for the development of diagnostic tools and vaccines. Currently, the molecular basis for P72 antigenicity remains largely unknown, and reports about antigenic epitopes of P72 are limited. In a previous study, a partial neutralizing mAb 135D4 was identified to recognize a conformational epitope on p72 between amino acid residues 400 and 404 (Borca et al., 1994). Subsequently, mAbs were prepared against a recombinant antigenic fragment, from amino acid (aa) 20-303, expressed in baculovirus, and four linear epitopes of P72 protein were identified (Heimerman et al., 2018). In the present study, IFA and western blotting were used to screen linear B-cell epitopes recognized by four mAbs against ASFV-P72. In order to determine the minimum linear epitope and key amino acid sites, we constructed shorter N- and C-truncated segments by deleting amino acids one by one and characterized by western blotting. To the best of our knowledge, we reported four new linear B cell epitope regions located on the P72 protein, namely ^31^SNIKNVNKSY^40^ recognized by 6C10, ^41^GKPDP^45^ recognized by 5F1, ^56^HLVHFNAH^63^ identified by 4E2 and ^185^ERLYE^189^ identified by 5H4.

African swine fever virus is an ancient evolutionary DNA virus with a wide range of genetic diversity and antigenic variability, which may affect the recognition of specific antigens in diagnosis and limit the effectiveness of ASFV vaccines (Malogolovkin and Kolbasov, 2019; Li et al., 2020). It is crucial for diagnostics and vaccines to select the conserved antigenic regions precisely. In this study, by comparing the P72 amino acid sequences from 106 ASFV isolates, we found that 3 out of 4 linear epitopes of P72 were completely conserved. In addition, location analysis indicated that all conserved linear epitopes were exposed on the surface of P72 protein, which may provide potential candidates for ASFV vaccine design and the development of diagnostic methods. The neutralization assays were performed on MA104 cell monolayers essentially as described previously (Gomez-Puertas and Escribano, 1997). As a result, none of the anti-P72 mAbs effectively neutralized ASFV (data not shown). The top of the P72 trimer is a propeller-like structure that extends to the outside of the virus (Wang et al., 2021), most likely the region where the neutralizing epitopes are located. However, none of the four linear epitopes identified in this experiment were at the top of the trimer, which can explain why these antibodies do not possess neutralizing activity. It also indicates that the neutralizing epitopes of the P72 protein are not the dominant epitopes.

In summary, we prepared anti-P72 mAbs based on recombinant baculoviruses co-expressing ASFV P72 and B602L. Three new anti-P72 mAbs were mapped, which can only recognize the conformational epitopes of P72 protein formed with the assistance of B602L. At the same time, four new linear B cell epitope regions located on the P72 protein were identified. Among them, epitopes ^31^SNIKNVNKSY^40^, ^41^GKPDP^45^ and ^185^ERLYE^189^ were highly conserved among diverse ASFV strains. Aside from scientific significance for understanding the basis of antibody-antigen interaction, these findings may provide a solid foundation for further investigations into the antigenic functions of ASFV P72 protein, and the development of diagnostics and effective ASFV vaccines.

## Data availability statement

The original contributions presented in this study are included in the article/Supplementary material, further inquiries can be directed to the corresponding author.

## Ethics statement

This animal study was reviewed and approved by Animal Care Committee of Yangzhou University in China.

## Author contributions

DY, RG, HS, JY, KQ, HC, and AQ performed to the material preparation, data collection, and analysis. DY wrote the first draft of the manuscript and revised by AQ. All authors contributed to the study conception, commented on previous versions of the manuscript, read and approved the final manuscript.
