# Supplementary material for: Identification of novel linear epitopes in P72 protein of African swine fever virus recognized by monoclonal antibodies
Source: Front Microbiol. 2022 Nov 2;13:1055820. doi: 10.3389/fmicb.2022.1055820 (PMC9666363; doi:10.3389/fmicb.2022.1055820)
Supplement: Supplementary file 1 [file Data_Sheet_1.docx]

Supplementary Table 1

Sequences of the primers used in this study.

| Primer names | Primer sequences (5’ - 3’) |
| --- | --- |
| P72 (EcoRI/XhoI) | F: CGGAATTCATGGCATCAGGAGGAGCTTTTTG |
|  | R: CCCTCGAGTTAGGTACTGTAACGCAGCACAGC |
| B602L (EcoRI/KpnI) | F: CGGAATTCATGGCAGAATTTAATATTGATGAGCTT |
|  | R: GGGGTACCTTACAATTCTGCTTTTGTATATAAAAT |
| M13 | F: GTTTTCCCAGTCACGAC |
|  | R: CAGGAAACAGCTATGAC |
| P1 | R: CCCTCGAGTTAAATGTTTTTAATAATAGGTAATGTGATCGGAT |
| P2 | R: CCCTCGAGTTAGCGAACGCGTTTTACAAAAAG |
| P3 | R: CCCTCGAGTTACTGATAGTATTTAGGGGTTTGAGGTC |
| P4 | R: CCCTCGAGTTAGATGCAAAATTTGCGCACAAG |
| P5 | R: CCCTCGAGTTAATGATGGCCCACCATATCAT |
| P4-1 | R: CCCTCGAGTTAGCAGTAGTAAACCAAGTTTCGG |
| P4-2 | R: CCCTCGAGTTACCAGTCATATCCGTTGCGAGGA |
| P4-3 | R: CCCTCGAGTTAGACATCCGAACTATATTC |
| P4-4 | R: CCCTCGAGTTAATTTCCATTTACATCGAATC |
| P4-5 | R: CCCTCGAGTTATTCATAAAGTCGTTCTCCG |
| P4-5-1 | R: CCCTCGAGTTATCGTTCTCCGGGGTATTCGCAG |
| P4-5-2 | R: CCCTCGAGTTAGGGGTATTCGCAGTAGTAAACC |
| P4-5-3 | R: CCCTCGAGTTAATAAAGTCGTTCTCCGG |
| P4-5-4 | R: CCCTCGAGTTAAAGTCGTTCTCCGGGGT |
| P5-1 | R: CCCTCGAGTTAAAACCCTACTGGAACATAAGGCTTAAAATGC |
| P5-2 | R: CCCTCGAGTTACACATTTTTAATGTTAGAGATCCTGCT |
| P5-1-1 | R: CCCTCGAGTTAATGCGCATTAAAATGCACCAAATGTG |
| P5-1-2 | R: CCCTCGAGTTATTCTTCGATTTGACTCAAAGTGGG |
| P5-1-3 | R: CCCTCGAGTTAGGGATCGGGTTTCCCATAACTT |
| P5-1-3-1 | R: CCCTCGAGTTATTTCCCATAACTTTTGTTCACA |
| P5-1-3-2 | R: CCCTCGAGTTAACTTTTGTTCACATTTTTAATG |
| P5-1-3-1-1 | R: CCCTCGAGTTACCCATAACTTTTGTTCACATTT |
| P5-1-3-1-2 | R: CCCTCGAGTTAATAACTTTTGTTCACATTTTTA |
| P5-1-3-3 | R: CCCTCGAGTTAATCGGGTTTCCCATAACTTTTG |
| P5-1-3-4 | R: CCCTCGAGTTAGGGTTTCCCATAACTTTTGTTC |
| P5-1-1-1 | R: CCCTCGAGTTAAAAATGCACCAAATGTGTTTCT |
| P5-1-1-2 | R: CCCTCGAGTTACAAATGTGTTTCTTCGATTT |
| P5-1-1-3 | R: CCCTCGAGTTACGCATTAAAATGCACCAAATGTGTTT |
| P5-1-1-4 | R: CCCTCGAGTTAATTAAAATGCACCAAATGTGTTTC |
| P6 | F: CGGAATTCATGTTGCTGAATAGCAGGATCTCTAAC |
| P6-1 | F: CGGAATTCATGATCTCTAACATTAAAAATGTGAACAA |
| P6-2 | F: CGGAATTCATGAATGTGAACAAAAGTTATGGGAAACC |
| P6-1-1 | F: CGGAATTCATGTCTAACATTAAAAATGTGAACAAAAG |
| P6-1-2 | F: CGGAATTCATGAACATTAAAAATGTGAACAAAAGTT |
| P6-3 | F: CGGAATTCATGTATGGGAAACCCGATCCCGA |
| P6-3-1 | F: CGGAATTCATGGGGAAACCCGATCCCGAAC |
| P6-3-2 | F: CGGAATTCATGAAACCCGATCCCGAACCCACTT |
| P7 | F: CGGAATTCATGACTTTGAGTCAAATCGAAGAAACAC |
| P7-1 | F: CGGAATTCATGGAAGAAACACATTTGGTGCATTT |
| P7-2 | F: CGGAATTCATGGTGCATTTTAATGCGCATTTTAAGCC |
| P7-1-1 | F: CGGAATTCATGGAAACACATTTGGTGCATTTTAAT |
| P7-1-2 | F: CGGAATTCATGACACATTTGGTGCATTTTAAT |
| P7-1-3 | F: CGGAATTCATGCATTTGGTGCATTTTAATGCGC |
| P7-1-4 | F: CGGAATTCATGTTGGTGCATTTTAATGCG |
| P8 | F: CGGAATTCATGCGAAACTTGGTTTACTACTGC |
| P8-1 | F: CGGAATTCATGTACTGCGAATACCCCGGAGAACG |
| P8-2 | F: CGGAATTCATGGGAGAACGACTTTATGAAAACG |
| P8-2-1 | F: CGGAATTCATGGAACGACTTTATGAAAACG |
| P8-2-2 | F: CGGAATTCATGCGACTTTATGAAAACGTAAG |
| P8-2-3 | F: CGGAATTCATGCTTTATGAAAACGTAAGATTCG |

The twenty-nine reverse primers (P1-P5) share the same forward primer P72-F. The twenty-one forward primers (P6-P8) share the same reverse primer P72-R.

Supplementary Table 2

Summary of mAbs reactivity to ASFV P72 fragments in IFA.

| Polypeptide fragment | Amino acid position | mAb | | | |
| --- | --- | --- | --- | --- | --- |
|  |  | 6C10 | 5F1 | 4E2 | 5H4 |
| P1 | 1-540 | + | + | + | + |
| P2 | 1-432 | + | + | + | + |
| P3 | 1-324 | + | + | + | + |
| P4 | 1-216 | + | + | + | + |
| P4-1 | 1-180 | + | + | + | - |
| P4-2 | 1-144 | + | + | + | - |
| P4-3 | 1-207 | + | + | + | + |
| P4-4 | 1-198 | + | + | + | + |
| P4-5 | 1-189 | + | + | + | + |
| P4-5-1 | 1-186 | + | + | + | - |
| P4-5-2 | 1-183 | + | + | + | - |
| P4-5-3 | 1-188 | + | + | + | - |
| P4-5-4 | 1-187 | + | + | + | - |
| P5 | 1-108 | + | + | + | - |
| P5-1 | 1-72 | + | + | + | - |
| P5-2 | 1-36 | - | - | - | - |
| P5-1-1 | 1-63 | + | + | + | - |
| P5-1-2 | 1-54 | + | + | - | - |
| P5-1-3 | 1-45 | + | + | - | - |
| P5-1-3-1 | 1-42 | + | - | - | - |
| P5-1-3-2 | 1-39 | - | - | - | - |
| P5-1-3-1-1 | 1-41 | + | - | - | - |
| P5-1-3-1-2 | 1-40 | + | - | - | - |
| P5-1-3-3 | 1-44 | + | - | - | - |
| P5-1-3-4 | 1-43 | + | - | - | - |
| P5-1-1-1 | 1-60 | + | + | - | - |
| P5-1-1-2 | 1-57 | + | + | - | - |
| P5-1-1-3 | 1-62 | + | + | - | - |
| P5-1-1-4 | 1-61 | + | + | - | - |
| P6 | 25-647 | + | + | + | + |
| P6-1 | 30-647 | + | + | + | + |
| P6-2 | 35-647 | - | + | + | + |
| P6-1-1 | 31-647 | + | + | + | + |
| P6-1-2 | 32-647 | - | + | + | + |
| P6-3 | 40-647 | - | + | + | + |
| P6-3-1 | 41-647 | - | + | + | + |
| P6-3-2 | 42-647 | - | - | + | + |
| P7 | 48-647 | - | - | + | + |
| P7-1 | 53-647 | - | - | + | + |
| P7-2 | 58-647 | - | - | - | + |
| P7-1-1 | 54-647 | - | - | + | + |
| P7-1-2 | 55-647 | - | - | + | + |
| P7-1-3 | 56-647 | - | - | + | + |
| P7-1-4 | 57-647 | - | - | - | + |
| P8 | 174-647 | - | - | - | + |
| P8-1 | 179-647 | - | - | - | + |
| P8-2 | 184-647 | - | - | - | + |
| P8-2-1 | 185-647 | - | - | - | + |
| P8-2-2 | 186-647 | - | - | - | - |
| P8-2-3 | 187-647 | - | - | - | - |

(+) positive; (-) negative.
